# Stem cell therapy for heart disease: truly beneficial?

**DOI:** 10.1007/s12471-014-0568-2

**Published:** 2014-06-13

**Authors:** E. E. van der Wall

**Affiliations:** Interuniversity Cardiology Institute of the Netherlands (ICIN), Netherlands Heart Institute, Catherijnesingel 52, P.O. Box 19258, 3501 DG Utrecht, the Netherlands

For over 10 years cardiac stem cell therapy has received a considerable amount of attraction, potential, and research money. Autologous bone marrow stem cell therapy has been reported to be safe and to substantially increase cardiac function [1–4]. However, this beneficial effect is not invariably the case as shown in 2011 by the Dutch HEBE trial directed by the Interuniversity Cardiology Institute of the Netherlands together with the Netherlands Heart Foundation (ICIN/NHS) [5–7]. More importantly, the present cardiac bone marrow stem cell research trials have differed in the effect sizes they reported, for reasons that are not fully understood. A recent meta-analysis published in the British Medical Journal (BMJ) on 28 April 2014, the so-called DAMASCENE study [8], did show that many of the most promising results in the field are illusive and that the potential benefits of stem cells to treat heart disease are probably far more modest than has been presumed until now. By pooling all available results from trials of bone marrow stem cells in patients with ischaemic heart disease or congestive heart failure, a Cochrane review found slight evidence to suggest a benefit for stem-cell therapy in those populations. In pooled results from smaller randomised trials it was shown that bone marrow stem cell treatment was associated with reduced mortality (RR 0.28, 95 % CI 0.14–0.53) and less hospitalisations for heart failure (RR 0.26, 95 % CI 0.07–0.94) at follow-up exceeding 1 year. However, the quality of the evidence was considered low, and no significant differences were seen in those outcomes with shorter follow-up. Additionally, it was commented that ‘*this research raises disturbing questions about ethics and research conduct and misconduct in a high-flying field*’ [9].

What did the BMJ study address in depth? Nowbar from the group of Francis (Imperial College, London, UK) investigated whether discrepancies in trials of use of bone marrow stem cells in patients with heart disease account for the variations in reported effect size in improvement of left ventricular function [8]. To that purpose, the authors selected randomised controlled trials that evaluated the effect of autologous bone marrow stem cells for heart disease on mean left ventricular ejection fraction (LVEF). The trials were examined for discrepancies and categorised into the following three types: 1) discrepancies in the design-for example, conflicting statements as to whether the study was randomised; 2) discrepancies in methods and baseline characteristics-for example, sample or subgroup sizes that could not be an integer number of patients; 3) discrepancies in results-for example, conflicts between tables and figures or impossible values. In total, over 600 discrepancies in 133 reports from 49 trials were found. One of the most important findings was a remarkable association between the number of discrepancies and the reported change in LVEF with bone marrow stem cell therapy, i.e. between the number of errors and the treatment effect. The five studies with no discrepancies showed no improvement in LVEF (−0.4 %), whereas the five studies with the largest number of discrepancies (>30) showed a significant improvement in LVEF (+7.7 %).

The authors had no straightforward explanation for the observed discrepancies. One possibility is that investigators might feel pressure for results to match expectations. One signal of a misguided desire to please is the phenomenon of directed editing of rounded percentages to force them to add up to 100 %. In reality, correctly rounded percentages often do not add up to 100 % when there are many categories. Secondly, exciting new treatments might be reported before full checking. One signal of this, in the neighbouring speciality of cardiomyocyte-derived stem cell therapy, is the insertion of the word ‘randomised’ into the title of the journal publication that was not present in the manuscript. There were seven controls in total, but after subtraction of the four who were not randomised and one who was randomised to stem cells but refused treatment, the number of randomised controls was only two. Thirdly, bone marrow stem cell therapy might be less effective when it is carried out in a rigidly standardised way. Institutions with less attention to detail might incorporate an unnoticed contaminant that enhances the effect of treatment, producing reports with more discrepancies. The final possibility is that in the reports with the fewest discrepancies, the LVEF effect might also have been measured with least error. If so, the true effect of bone marrow stem cells on ejection fraction is zero.

What lessons can be learned from this meta-analysis. The authors strongly suggest that the following useful information can be drawn for the design of future trials of bone marrow stem cells: 1) prior registration on a public clinical trial registry should become universal and will be helpful in distinguishing unambiguously between trials that were multiply published or merely identical by coincidence; 2) reports should include a spreadsheet of all the data used for construction of the tables, so that incorrect values could be more easily identified (disclosing the individual patient data could help to correct more errors); 3) it is important for studies, when solely relying on changes in LVEF as an endpoint, to be properly designed to resist error and to have adequate sample size to ‘beat’ the effects of biological variability. LVEF is a changeable variable, which in some modalities is easily manipulated innocently by clinicians who have prior beliefs on what a realistic value should be for a particular patient. Sample size planning can sometimes be erroneously omitted when clinicians are enthusiastic to demonstrate the effectiveness of a treatment seen as exciting.

These astonishing findings call into question the validity of cardiac stem cell therapy; is it as beneficial as has been proclaimed? Recently two important papers from the stem cell expert group led by Piero Anversa (Boston, USA), one published in Circulation in 2012 [10] and one in the Lancet in 2011 [11], have been discredited as a result of an ongoing investigation at Harvard Medical School and Brigham and Women’s Hospital in Boston. As a result of this investigation, the Circulation paper was recently (22 April 2014) retracted by the American Heart Association because ‘*the data are sufficiently compromised that a retraction is warranted’* [10]. The Lancet, which published the SCIPIO study in 2011 (also from the Boston group) [11, 12], issued a formal Expression of Concern in March of this year. Francis and colleagues had already issued a warning about this in 2013 [13], when they expressed devastating critiques of multiple papers from the German research group directed by Strauer [14], and the C-CURE study published in the Journal of the American College of Cardiology (JACC) [15].

To summarise, the current meta-analysis in BMJ clearly shows that any reports of trials of bone marrow stem cell therapy contain factual discrepancies. Avoiding discrepancies is difficult but is important because discrepancy count is related to effect size. Trials with over 30 discrepancies report a large effect size, whereas trials with fewer discrepancies have found progressively smaller effect sizes, culminating in discrepancy-free trials reporting an effect size of zero. The mechanism is still unknown but should be explored in the design of future bone marrow stem cell trials. Consequently, there is an obvious need for large-scale, adequately powered studies with well-defined participant cohorts and long-term follow-up to confirm (or refute) the beneficial effects of bone marrow stem cells in terms of improved cardiac function, less hospitalisations and reduced mortality.
